# Supplementary material for: The influence of non-family members in top management teams on research and development investment: Evidence from Chinese family firms
Source: PLoS One. 2021 Oct 8;16(10):e0258200. doi: 10.1371/journal.pone.0258200 (PMC8500433; doi:10.1371/journal.pone.0258200)
Supplement: Attachment — Submitted filename: PLOS ONE_Reviewer Comments_2021.docx [file pone.0258200.s002.docx]

Manuscript title: The influence of non-family members in top management teams on research and development investment: Evidence from Chinese family firms

1. Originality: Does the paper contain new and significant information adequate to justify publication?

This paper investigates the R&D investment propensity of family firms from the perspective of non-family members’ participation in TMTs. Based on the upper echelons and the socioemotional wealth theory, this paper incorporated the non-economic goals that influence strategic decisions in family firms into the analytical framework. The paper is very interesting and is expected to contribute to the readers in emerging and established markets. The suggestions are also timely and appropriate. Overall, it is a good piece of work.  However, I have some suggestions which could help to improve on the quality of your paper especially in your introduction section. Specifically, you need to support your introduction with a few more (e.g., 2-3) up-to-date journal articles as some of them are outdated.

2. Relationship to literature: Does the paper demonstrate an adequate understanding of the relevant literature in the field and cite an appropriate range of literature sources? Is any significant work ignored?

The literature review is comprehensive with good perspectives to each of the points and captured the latest works in the related areas. Additionally, the hypotheses development is well written. Well done! May I suggest including a conceptual framework for the reader’s imagination on the build-up of the hypotheses.

3. Methodology: Is the paper’s argument built on an appropriate base of theory, concepts, or other ideas? Has the research or equivalent intellectual work on which the paper is based been well designed? Are the methods employed appropriate?

They are acceptable based on the purpose of study.

4. Results: Are results presented clearly and analyzed appropriately? Do the conclusions adequate tie together the other elements of the paper?

Yes, the results are presented and analyzed appropriately

5. Implications for research, practice and or society. Does the paper identify clearly between any implications for research, practice and or society? Does the paper bridge the gap between theory and practice? How can the research be used in practice, in teaching, to influence public policy, in research? What is the impact upon society? Are these implications consistent with the findings and conclusion of the paper?

They are well written! Well done!

6. Quality of communication: Does the paper clearly express its case, measured against the technical language of the field and the expected knowledge of the journal’s readership? Has attention been paid to the clarity of expression and readability, such as sentence structure, jargon use, acronyms, etc

The reviewer spotted some grammatical errors in the paper; please do thorough grammatical checking to the manuscript to improve the readability of this study.

7. References

The references are not in line with PLOS ONE. May I suggest changing it to the required references if the manuscript is accepted for publication.

Reviewer recommendation to Editor-in-Chief

The paper clearly establishes itself that there is new and exciting knowledge from this study. The reviewer would recommend this paper as: Accepted with minor amendments.
