# Supplementary material for: The influence of non-family members in top management teams on research and development investment: Evidence from Chinese family firms
Source: PLoS One. 2021 Oct 8;16(10):e0258200. doi: 10.1371/journal.pone.0258200 (PMC8500433; doi:10.1371/journal.pone.0258200)
Supplement: Attachment — Submitted filename: Response to Reviewers.doc [file pone.0258200.s003.doc]

Response to comments

Thank you for your letter and the reviewers’ comments concerning our manuscript entitled “**The influence of non-family members in top management teams on research and development investment: Evidence from Chinese family firms**” (Manuscript ID: PONE-D-21-15220). Those comments are all valuable and very helpful for revising and improving our paper, as well as the important guiding significance to our researches. We have studied comments carefully and have made correction which we hope meet with approval. Revised portion are marked in red in revised paper. The main corrections in the paper and the responds to the reviewer’s comments are as follows:

**Section 1: Reviewer's Responses to Questions**

(1) Is the manuscript technically sound, and do the data support the conclusions?

The manuscript must describe a technically sound piece of scientific research with data that supports the conclusions. Experiments must have been conducted rigorously, with appropriate controls, replication, and sample sizes. The conclusions must be drawn appropriately based on the data presented.

Reviewer #1: Yes

Reviewer #2: Yes

**Response:** Thank you for reviewers' support and responses.

(2) Has the statistical analysis been performed appropriately and rigorously?

Reviewer #1: Yes

Reviewer #2: Yes

**Response:** Thank you for reviewers' support and responses.

(3) Have the authors made all data underlying the findings in their manuscript fully available?

Reviewer #1: Yes

Reviewer #2: Yes

**Response:** Thank you for reviewers' support and responses.

(4) Is the manuscript presented in an intelligible fashion and written in standard English?

Reviewer #1: Yes

Reviewer #2: No

**Response:** Thank you for reviewers' support and responses.

**Responses to Reviewer #2:** The authors have thoroughly checked the manuscript for grammar to improve the readability of this study. These changes are highlighted in red in text.

**Section 2: Response to Comments for Reviewers**

**Reviewer #1:** You mentioned in the ethics declaration that NA but clearly this study warranted an ethics clearance. Do kindly look into this matter as it involves humans. Also the referencing needs to be aligned with PLOS ONE criteria. Minor typo errors are encountered in the paper. Updating of a few more relevant and up-to-date journals in the Introduction section will be good and needed.

**Response:** Thank you for reviewers' support and responses. First, we have carefully considered the issue of ethical clearance according to reviewer’s suggestion. Therefore, we have revised the selection of ethics declaration in the process of submitting a revised version. Second, we have revised the references to be consistent with PLOS ONE criteria. Also, we have updated some more relevant and up-to-date journals in the full text, especially in the Introduction section. Finally, we have performed a detailed check of the manuscript to improve the readability of this study. These changes above are highlighted in red in text.

(1) Originality: Does the paper contain new and significant information adequate to justify publication? This paper investigates the R&D investment propensity of family firms from the perspective of non-family members’ participation in TMTs. Based on the upper echelons and the socioemotional wealth theory, this paper incorporated the non-economic goals that influence strategic decisions in family firms into the analytical framework. The paper is very interesting and is expected to contribute to the readers in emerging and established markets. The suggestions are also timely and appropriate. Overall, it is a good piece of work. However, I have some suggestions which could help to improve on the quality of your paper especially in your introduction section. Specifically, you need to support your introduction with a few more (e.g., 2-3) up-to-date journal articles as some of them are outdated.

**Response:** Thank you for reviewers' support and responses. We have updated thirteen journal articles according to reviewer’s suggestion. These changes above are highlighted in red in text.

(2) Relationship to literature: Does the paper demonstrate an adequate understanding of the relevant literature in the field and cite an appropriate range of literature sources? Is any significant work ignored? The literature review is comprehensive with good perspectives to each of the points and captured the latest works in the related areas. Additionally, the hypotheses development is well written. Well done! May I suggest including a conceptual framework for the reader’s imagination on the build-up of the hypotheses.

**Response:** Thank you for reviewers' support and responses. We have added a conceptual framework in the Hypotheses section according to reviewer’s suggestion. These changes are highlighted in red in text. The details are as follows:

Based on the characteristics of Chinese family firms, this study examined the impact of importing outsiders into TMTs on the strategic management and innovation goal intention of family firms. In addition, considering the influence of family members on management decisions, this study divided the affective goals of family members into three dimensions: control and influence, status perception, and social responsibility. It also analyzed the moderating effects of these emotional goals on the relationship between the involvement of non-family members in TMT and R&D investment. Fig. 1 shows a visual representation of the core constructs in our study and the proposed relationships among them.

Fig 1. The conceptual framework of hypotheses

(3) Results: Are results presented clearly and analyzed appropriately? Do the conclusions adequate tie together the other elements of the paper? Yes, the results are presented and analyzed appropriately.

**Response:** Thank you for reviewers' support and responses.

(4) Implications for research, practice and or society. Does the paper identify clearly between any implications for research, practice and or society? Does the paper bridge the gap between theory and practice? How can the research be used in practice, in teaching, to influence public policy, in research? What is the impact upon society? Are these implications consistent with the findings and conclusion of the paper? They are well written! Well done!

**Response:** Thank you for reviewers' support and responses.

(5) Quality of communication: Does the paper clearly express its case, measured against the technical language of the field and the expected knowledge of the journal’s readership? Has attention been paid to the clarity of expression and readability, such as sentence structure, jargon use, acronyms, etc. The reviewer spotted some grammatical errors in the paper; please do thorough grammatical checking to the manuscript to improve the readability of this study.

**Response:** Thank you for reviewers' support and responses. The authors have thoroughly checked the manuscript for grammar to improve the readability of this study. These changes are highlighted in red in text.

(6) References. The references are not in line with PLOS ONE. May I suggest changing it to the required references if the manuscript is accepted for publication.

**Response:** Thank you for reviewers' support and responses. We have revised the references to be consistent with PLOS ONE criteria.

**Reviewer #2:** I am glad to review and assess this interesting article, entitled, The influence of non-family members in top management teams on research and development investment: Evidence from Chinese family firms. The organization of this article is good and satisfactory. The Introduction section and methodology portions are adequate. I suggest the authors improve the introduction & Methodology section by adding some latest articles' citations to enhance the work quality and also concise this part. Also, Improve the Conclusion part as well. Overall, the manuscript is a good piece of work. I recommend that authors do a little more work and add the latest literature to support the study, as suggested. The English level is good and smooth, e.g., the language standard, specifically the grammar, of sufficient quality to meet scientific merit for publication. I accept this manuscript after minor revision, as I have recommended.

**Response:** Thank you for reviewers' support and responses. In the Introduction and Methodology section, we have added citations to some recent articles to improve the work quality and have simplified these sections. Moreover, in order to enhance the value of considering the country context of our study, we compared findings with those from studies in different countries, which reflects the particularity of family firms in Chinese context. These changes are highlighted in red in text.

**Section 3: Response to Additional Requirements**

(1) Please ensure that your manuscript meets PLOS ONE's style requirements, including those for file naming.

**Response:** We have revised the manuscript according to the style requirements of PLOS ONE.

(2) Please include additional information regarding the survey or questionnaire used in the study and ensure that you have provided sufficient details that others could replicate the analyses. For instance, if you developed a questionnaire as part of this study and it is not under a copyright more restrictive than CC-BY, please include a copy, in both the original language and English, as Supporting Information

**Response:** This study did not develop a questionnaire. The original data in the manuscript came from the sample survey database of Chinese private enterprises in 2010 (https://cpes.zkey.cc/index.jsp).

(3) In your Data Availability statement, you have not specified where the minimal data set underlying the results described in your manuscript can be found.

Response: We have supported our minimal dataset as a supporting Information file, which is from Chinese Private Enterprise Survey (CPES).

(4) Please include captions for your Supporting Information files at the end of your manuscript, and update any in-text citations to match accordingly.

**Response:** We have added the title of the supporting information files at the end of the manuscript. These changes are highlighted in red in text.

Special thanks for reviewers’ and editor’s comments. We tried our best to improve the manuscript and made some changes in the manuscript. These changes will not influence the content of the paper and the changes were highlighted in red in text.

We appreciate for Editors/Reviewers’ warm work earnestly, and hope that the correction will meet with approval.

Once again, thank you very much for your comments and suggestions.

With all best wishes.
