# Supplementary material for: The influence of non-family members in top management teams on research and development investment: Evidence from Chinese family firms
Source: PLoS One. 2021 Oct 8;16(10):e0258200. doi: 10.1371/journal.pone.0258200 (PMC8500433; doi:10.1371/journal.pone.0258200)
Supplement: Attachment — Submitted filename: Response to Reviewers.doc [file pone.0258200.s004.doc]

Response to comments

Thank you for your letter and the comments concerning our manuscript entitled “**The influence of non-family members in top management teams on research and development investment: Evidence from Chinese family firms**” (Manuscript ID: PONE-D-21-15220). The main corrections in the paper are as follows:

(1) First, as one reviewer pointed out, this study involves humans (survey). Please clarify this issue or provide approval for human subjects research by an institutional review board (IRB) or equivalent ethics committee(s) if necessary.

**Response:** Thank you for your support and response. We have revised the selection of ethics declaration in the process of submitting a revised version and added this statement at the beginning of the Methodology section of manuscript. These changes are highlighted in red in text. The details are as follows: The names of the institutional review board that approved the questionnaire survey are the Institute of Sociology Chinese Academy of Social Sciences and All-China Federation of Industry and Commerce, which issued a statement approving this research (http://ssm.cssn.cn/sjzy/201609/t20160914_3202846.shtml). Moreover, the survey data was analyzed anonymously.

(2) Second, reference needs to be updated reflecting the latest literature.

**Response:** Thank you for your support and response. We have updated seventeen journal articles according to the suggestion. These changes are highlighted in red in text. The details are as follows:

2.	Calabro A, Vecchiarini M, Gast J, Campopiano G, De Massis A, Kraus S. Innovation in Family Firms: A Systematic Literature Review and Guidance for Future Research. International Journal of Management Reviews. 2019;21(3):317-355. doi: 10.1111/ijmr.12192.

4.	Classen N, Carree M, Van Gils A, Peters B. Jiang F, Shi W, Zheng X. Board chairs and R&D investment: Evidence from Chinese family-controlled firms. Journal of Business Research. 2020;112(5):109-118. doi: 10.1016/j.jbusres.2020.02.026.

6.	Dieguez-Soto, J, Martinez-Romero, MJ. Family Involvement in Management and Product Innovation: The Mediating Role of R&D Strategies. Sustainability. 2019;11(7):1-24. doi: 10.3390/su11072162.

7.	Zulfiqar M, Zhang R, Khan N. Behavior Towards R&D Investment of Family Firms CEOs: The Role of Psychological Attribute. Psychology Research and Behavior Management. 2021;14:595-620. doi: 10.2147/PRBM.S306443.

19.	Wu T, Wu YJ, Tsai H, Li Y. Top Management Teams' Characteristics and Strategic Decision-Making: A Mediation of Risk Perceptions and Mental Models. Sustainability. 2017;9(12):1-15. doi: 10.3390/su9122265.

21.	De Massis A, Eddleston KA, Rovelli P. Entrepreneurial by Design: How Organizational Design Affects Family and Non-family Firms' Opportunity Exploitation. Journal of Management Studies. 2021;58(1):27-62. doi: 10.1111/joms.12568.

25.	Prokop V, Stejskal J, Klimova V, Zitek V. The role of foreign technologies and R&D in innovation processes within catching-up CEE countries. PLoS One. 2021;16(4):e0250307. doi: 10.1371/journal.pone.0250307.

33.	Yang B, Nahm A, Song Z. Succession, political resources, and innovation investments of family businesses: Evidence from China. Managerial and Decision Economics. 2021. doi: 10.1002/mde.3385.

35.	Ge Y, Kong X, Dadilabang G, Ho K. The effect of Confucian culture on household risky asset holdings: Using categorical principal component analysis. International Journal of Finance & Economics. 2021. doi: 10.1002/ijfe.2452.

39.	Carmen Diaz-Fernandez M, Rosario Gonzalez-Rodriguez M, Simonetti B. The role played by job and non-job-related TMT diversity traits on firm performance and strategic change. Management Decision. 2016;54(5):1110-1139. doi: 10.1108/md-10-2015-0464.

78.	Newbert S, Craig JB. Moving Beyond Socioemotional Wealth: Toward a Normative Theory of Decision Making in Family Business. Family Business Review. 2017;30(4):339-346. doi: 10.1177/0894486517733572.

79.	El Akremi A, Gond J-P, Swaen V, De Roeck K, Igalens J. How Do Employees Perceive Corporate Responsibility? Development and Validation of a Multidimensional Corporate Stakeholder Responsibility Scale. Journal of Management. 2018;44(2):619-657. doi: 10.1177/0149206315569311.

82.	Li X. The effectiveness of internal control and innovation performance: An intermediary effect based on corporate social responsibility. PLoS One. 2020;15(6):e0234506. doi: 10.1371/journal.pone.0234506.

83.	Alpkan L, Gülsoy T. Introduction to the Special Issue on Strategic Management in Emerging Economies: Innovation, Corporate Social Responsibility, and Sustainable Management. International Journal of Innovation and Technology Management. 2019;16(4):1-4. doi: 10.1142/S0219877019020012.

93.	Mehrabi H, Coviello N, Ranaweera C. When is top management team heterogeneity beneficial for product exploration? Understanding the role of institutional pressures. Journal of Business Research. 2021;132:775-786. doi: 10.1016/j.jbusres.2020.10.057.

95.	Salah OH, Yusof ZM, Mohamed H. The determinant factors for the adoption of CRM in the Palestinian SMEs: The moderating effect of firm size. PLoS One. 2021;16(3):e0243355. doi: 10.1371/journal.pone.0243355.

101.	Lee K, Makri M, Scandura T. The Effect of Psychological Ownership on Corporate Entrepreneurship: Comparisons Between Family and Nonfamily Top Management Team Members. Family Business Review. 2019;32(1):10-30. doi: 10.1177/0894486518785847.

(3) There are typo and errors in the paper. Please proof edit the manuscript before submission.

**Response:** Thank you for your support and response. We have performed a detailed check of the manuscript to improve the readability of this study. These changes are highlighted in red in text.

Special thanks for reviewers’ and editor’s comments. We tried our best to improve the manuscript and made some changes in the manuscript. These changes will not influence the content of the paper and the changes were highlighted in red in text.

We appreciate for Editors/Reviewers’ warm work earnestly, and hope that the correction will meet with approval.

Once again, thank you very much for your comments and suggestions.

With all best wishes.
